# Feasibility study of an integrated stroke self-management programme: a cluster-randomised controlled trial

**DOI:** 10.1136/bmjopen-2015-008900

**Published:** 2016-01-06

**Authors:** Fiona Jones, Heather Gage, Avril Drummond, Ajay Bhalla, Robert Grant, Sheila Lennon, Christopher McKevitt, Afsane Riazi, Matthew Liston

**Affiliations:** 1 Faculty of Health, Social Care and Education, Kingston University and St George's, University of London, London, UK; 2 School of Economics, University of Surrey, Surrey, UK; 3 School of Health Sciences, University of Nottingham, Nottingham, UK; 4 Division of Health and Ageing, Guy's & St Thomas NHS Foundation Trust, London, UK; 5 School of Health Sciences, Flinders University, Daw Park, South Australia, Australia; 6 Division of Health & Social Care Research, Guy's and St Thomas’ NHS Foundation Trust, King's College London, London, UK; 7 Department of Psychology, Royal Holloway University of London, Surrey, UK; 8 School of Science and Health, University of Western Sydney, Sydney, Australia

**Keywords:** REHABILITATION MEDICINE, PUBLIC HEALTH

## Abstract

**Objectives:**

To test the feasibility of conducting a controlled trial into the effectiveness of a self-management programme integrated into stroke rehabilitation.

**Design:**

A feasibility cluster-randomised design was utilised with stroke rehabilitation teams as units of randomisation.

**Setting:**

Community-based stroke rehabilitation teams in London.

**Participants:**

78 patients with a diagnosis of stroke requiring community based rehabilitation.

**Intervention:**

The intervention consisted of an individualised approach to self-management based on self-efficacy. Clinicians were trained to integrate defined self-management principles into scheduled rehabilitation sessions, supported by a patient-held workbook.

**Main outcomes measures:**

Patient measures of quality of life, mood, self-efficacy and functional capacity, and health and social care utilisation, were carried out by blinded assessors at baseline, 6 weeks and 12 weeks. Fidelity and acceptability of the delivery were evaluated by observation and interviews.

**Results:**

4 community stroke rehabilitation teams were recruited, and received a total of 317 stroke referrals over 14 months. Of these, 138 met trial eligibility criteria and 78 participants were finally recruited (56.5%). Demographic and baseline outcome measures were similar between intervention and control arms, with the exception of age. All outcome measures were feasible to use and clinical data at 12 weeks were completed for 66/78 participants (85%; 95% CI 75% to 92%). There was no significant difference in any of the outcomes between the arms of the trial, but measures of functional capacity and self-efficacy showed responsiveness to the intervention. Observation and interview data confirmed acceptability and fidelity of delivery according to predetermined criteria. Costs varied by site.

**Conclusions:**

It was feasible to integrate a stroke self-management programme into community rehabilitation, using key principles. Some data were lost to follow-up, but overall results support the need for conducting further research in this area and provide data to support the design of a definitive trial.

**Trial registration number:**

ISRCTN42534180.

**Strengths and limitations of this study**

- This is the first feasibility trial of an integrated approach to stroke self-management; study recruitment and findings support further research to test the intervention in a definitive trial.
- Community stroke rehabilitation teams had a high turnover of staff, and training needs were higher than anticipated, but intervention fidelity was maintained.
- The intervention requires some modification to be more accessible for those patients with cognitive and communication impairments, and for those having less than six sessions of rehabilitation.

## Introduction

Significant improvements have been made in the quality and effectiveness of acute stroke care across the developed world.^1–3^ But variation in the availability of post-hospital rehabilitation and support for self-managed activities still exists,^3^ ^4^ and the prevalence of mood disorders and social isolation post-stroke remains high.^5–7^ As the overall global burden of stroke increases,^1^ expenditure on the direct and indirect costs of stroke care is likely to rise, and in the UK this currently constitutes 5% of the total National Health Service (NHS) budget (£8 billion).^8^ Stroke and associated care models are still largely defined by acute medical ideologies and there is an inequity in attention to address long-term psychological and social sequelae.^9–11^ Arguably, unmet needs post-stroke could be exacerbated by care models that foster dependency on professional expertise in the acute stages, combined with a paucity of programmes to facilitate coping and self-management in the longer term.

One alternative to existing care models is the use of self-management programmes (SMPs) that build on growing evidence from systematic reviews in other long-term conditions.^12–15^ SMPs can be ‘provider-based’, delivered by healthcare professionals integrated into usual care, or ‘patient-based’, when supplied in addition to care through group or individual education.^13–16^ Broadly, self-management focuses on those actions individuals and others take to mitigate the effects of a long-term condition, and to maintain the best possible quality of life.^12–14^ The variation in programmes makes it difficult to compare outcomes, but effective SMPs can improve mental well-being and quality of life, and reduce hospital readmission rates.^12–16^

The UK National Stroke Strategy, in 2007, advocated self-management initiatives to address long-term unmet needs,^17^ and national guidance recommends that all patients be offered training in self-management skills.^18^ Research to develop and evaluate stroke SMPs mainly comprises feasibility and phase II trials of group-based programmes, which, while demonstrating some impact on function, mood and quality of life,^9^ ^19^ ^20^ will not be accessible for certain patients with communication and cognitive impairments.^21^ We hypothesised that an individualised stroke self-management intervention that can be integrated into existing rehabilitation may extend the reach to more patients.^11^ ^19^ ^22^

Following the Medical Research Council Framework for the Development and Evaluation of Complex Interventions,^23^ several studies have been conducted to inform the development of an individualised SMP.^22^ ^24^ The Bridges stroke SMP is based on social cognition theory and self-efficacy,^25^ ^26^ and incorporates a patient-held workbook used by rehabilitation professionals to support self-management skills. Studies have demonstrated preliminary proof of concept and feasibility when provided in addition to rehabilitation.^22^ ^24^ However, an SMP delivered in addition to routine stroke rehabilitation has cost and time implications, especially when utilising an individualised approach. If the same programme could be integrated into existing rehabilitation, this may offer a solution that could be both clinically valuable and cost-effective.

The aim of the study was to test the feasibility of conducting a cluster-randomised controlled trial into the effectiveness of a stroke SMP (Bridges) integrated into community rehabilitation. We aimed to evaluate key trial parameters such as recruitment and retention of participants, randomisation, utility and sensitivity of outcome measures, levels of missing data and preliminary indications of effectiveness to inform calculation of a sample size for powering a full trial. An estimation of resources required to deliver the intervention and indications of likely cost-effectiveness were also investigated. Fidelity of the intervention delivery, training required and acceptability of the intervention to patients and clinicians were evaluated.

## Methods

### Design

A feasibility cluster-randomised design with a nested process evaluation was utilised with community stroke rehabilitation (CSR) teams as units of randomisation. Sites were eligible if they comprised multiprofessional teams with stroke specialist skills delivering post-hospital rehabilitation according to quality criteria set out in UK National Clinical Guidelines for Stroke.^18^ Current models of CSR in the UK provide rehabilitation by therapists (occupational therapists, physiotherapists and speech and language therapists) and non-professional support workers in patients’ homes.

### Selection of sites

Twenty-one CSR teams from outer and inner London boroughs with ethnically and socially diverse populations were sent information about the study via a group email used for a pan-London Stroke Rehabilitation Network. Six teams agreed to take part and four teams were selected as they had not taken part in any previous self-management training in the previous 12 months, and met all other eligibility criteria. Team consent was obtained from the lead clinician acting as a cluster guardian.

### Randomisation

Allocation of CSR teams to either an intervention or control cluster was carried out once teams had been recruited and given consent to participate by a local clinical trials unit via simple randomisation at 1:1 ratio without matching.

### Intervention

Intervention site teams undertook training on theory, research and practical application of the Bridges SMP. Training delivery in intervention sites adhered to a predetermined protocol based on seven key principles of the SMP; these were developed through previous research and in consultation with key stakeholders (table 1).^22^ ^24^

**Table 1:** Seven key principles of the Bridges stroke self-management programme

Key principle	An example of what might be observed to demonstrate use of key principle
**Problem solving** Not being given solutions but encouraged to come up with ideas and strategies	Clinician reminds patient about how the patient has earlier found ways around a problem or challenge, for example, “I remember when you had to work really hard to do ‘x’—how did you manage that, is there any way you can use the same skills now?”
	
**Reflection** Attributing changes and progress to personal effort/not skills of therapist	Clinician encourages regular reflection in workbook to capture changes and monitor how progress is being made, for example, highlighting the value of reflecting on progress: “It will help to have a reminder about all the things you have managed to do, however small”
	
**Goal setting** Avoiding therapy-led goals, encouraging small steps for mastery experiences and longer term goals	Patient is encouraged to think of small things they could do towards their goal, instead of being discouraged from an ‘unrealistic target’, for example, “What's a small thing you could do this week that might help you towards that?”
	
**Accessing resources** Using resources available to achieve personal goals	Clinician uses open style coaching questions, for example, “What support could you use to help you get to that?”
	
**Self-discovery** Finding out new ways of doing things and trying out different activities	Clinician asks about the ways the patient managed to do challenging things before their stroke and what strategies have worked for them previously, for example, clinician is heard discussing the need to take some risks, and try things out and the benefit to learning about what is possible
	
**Activity** Encouraging activity, however small	Clinician asks what they have managed to do in the last week, what they are most pleased with in terms of their activity, for example, “What have you managed to do in the past week that you are really pleased about?”
	
**Knowledge** Knowledge about stroke, but also about self	Clinician explores what the patient knows about their stroke, what they would like to know and any concerns that patient feels might be hampering rehabilitation, for example, “Are there any things that you are worried might be affecting your rehab? Is there one small thing we can work towards that might help?”

The Bridges SMP aimed to be distinct from routine stroke rehabilitation provision in two main ways:

1. One-to-one rehabilitation sessions using seven principles integrated into each therapy session to support self-management activities.
2. A stroke workbook that included vignettes, activities, ideas and solutions from other stroke survivors for successful self-management, and space to record and reflect on goals and progress.

### Recruitment

Consecutive patients with stroke referred for CSR were screened by the community rehabilitation teams, recruited within 2 weeks of referral to the CSR team and consented by research staff not blinded to allocation. Patients were eligible if they had a confirmed diagnosis of stroke and could follow a two-stage command such as close your eyes and nod your head, and read simple text and/or have a carer to assist. Criteria were informed by previous research.^22^ ^24^

Stroke participants allocated to the intervention clusters were introduced to the stroke workbook and the seven key principles of self-management by the therapist integrated into existing CSR sessions. Participants in control sites received CSR as usual, which included access to physiotherapy, occupational therapy, and speech and language therapy, if required.

### Sample size

As this was a feasibility study, a prospective sample size calculation was not conducted. We aimed to recruit 80 stroke participants across the four sites over 14 months, which appeared realistic given the teams’ referral rates.

### Assessments

Data were collected in participants’ homes by research assessors blinded to group allocation. Clinical outcomes were collected at baseline (within 2 weeks from starting rehabilitation), 6 weeks and 12 weeks after baseline.

### Feasibility, fidelity and acceptability

The feasibility of recruiting and retaining participants was assessed from study records, and characteristics of those who were not eligible, consent and completion rates were analysed. Participants’ age, sex, social support, socioeconomic status and medical history, were described and compared between groups, to test randomisation.

Fidelity and acceptability of the delivery of the intervention were determined by observing a proportion of rehabilitation sessions, using a checklist to record patient and professional activities and behaviours against each principle component of the SMP. The checklist was piloted to enable a method to compare self-management support delivered in intervention and control sites that could be used in a larger trial. Patients and clinicians were interviewed in each site, to compare their experiences and understanding of self-management; those in the intervention site were specifically asked about the feasibility and acceptability of using self-management strategies and workbook.

### Clinical outcomes

Clinical measures found sensitive to change in previous self-management trials and validated in stroke populations were utilised,^9^ ^20^ ^24^ and included the Stroke and Aphasia Quality of Life (SAQOL) scale,^27^ Nottingham Extended Activities of Daily Living Scale (NEADL) of functional ability,^28^ Stroke Self-Efficacy Questionnaire (SSEQ),^29^ ^30^ and Hospital Anxiety and Depression Scale (HAD).^31^ The Medical Outcomes Trust's Short Form 12 (SF-12) was included to provide a generic measure health-related quality of life.^32^ Ease of data capture and levels of missing data were assessed for each outcome measure.

Although the study was not powered, a statistical analysis was conducted to gain a preliminary indication of effectiveness and of the feasibility of such analysis. The analysis enabled an assessment of the sensitivity of different outcome measures and provided a basis for a sample size calculation for the full trial.

### Statistical analysis

Considering feasibility, we compared levels of missing data between intervention and control sites using Fisher's exact test. In order to adjust for age, a multilevel regression model was fitted to each clinical outcome. This is a common approach to cluster-randomised clinical trials, and utilises all data, even if a participant is missing some. Group allocation was purely on the basis of site, forming an intention-to-treat analysis. Interparticipant variability was represented as a random intercept, and age, time and group allocation were included as fixed effects. Group differences were quantified at 6 and 12 weeks, and a composite null hypothesis that both were equal to zero was assessed by Wald tests. This represents no mean difference between groups in how the outcomes change over time. These analyses were conducted in Stata V.11.2 software (StataCorp, College Station, Texas, USA), using command ‘xtmixed’.

Sample size calculations for a future trial were calculated using Stata software (command ‘sampsi’), assuming SDs observed in this study for NEADL and SAQOL, 80% power requirement and a range of putative minimum clinically important differences (MCIDs): NEADL from 2 to 5 in steps of 0.1, and SAQOL from 0.1 to 0.5 in steps of 0.01.

### Economic analysis

To estimate the resources involved in delivering stroke rehabilitation in each site, data were collected at individual patient level from therapist records on the number of CSR sessions, and face-to-face contact time in minutes. Physical resources were converted to costs using validated national unit costs, in British pounds, 2012.^33^ Costs associated with patient-related non-face-to-face time was calculated under three alternative assumptions. Total costs were compared across sites.

The feasibility of capturing health and social care utilisation from participants was assessed using a bespoke self-report questionnaire administered to participants at weeks 6 and 12. Items included contacts with general practitioner (GP), practice nurse or other professionals, social care, and help from family and friends. The purpose was to explore if use of SMP reduced demands on other services, compared with the control group. EuroQol (EQ-5D) health state utility weights, using a published transformation^33^ of the SF-12 profile measure of quality of life, was to be tested for deriving quality-adjusted life year (QALY) gains.

## Results

### Intervention fidelity and acceptability

Overall, 63 occupational therapists, physiotherapists, speech and language therapists, and rehabilitation support workers, received training. This number was higher than expected because of clinician turnover.

The feasibility of monitoring intervention fidelity was evaluated through observations of a consecutive sample of 14 participants (18%, control n=7, intervention n=7). The checklist was feasible to use and identified whether CSR incorporated behaviours and activities relating to core self-management principles. Clinicians in the intervention sites showed use of between five and seven self-management principles, whereas those in the control site showed evidence of using two or less.

A consecutive sample of patients (n=23) were interviewed and focus groups were carried out with all clinicians (n=34) including occupational therapists, physiotherapists, speech and language therapists, and rehabilitation support workers across sites, at the end of the trial, to explore intervention feasibility and acceptability. Findings showed shared understanding of self-management in patients and clinicians within the intervention clusters, which reflected the underlying principles of the SMP and will be reported more fully elsewhere.

### Feasibility

*Recruitment rates*: Four sites were recruited from six CSR teams in London that expressed an interest and were eligible; excluded sites had either previously taken part in self-management training or were likely to undergo significant reorganisation during the trial period of 22 months. Participant recruitment occurred between July 2012 and August 2013, 138/317 patients (44%) were eligible to participate across four sites. Recruitment took 14 months, which was longer than the anticipated 10 months. This was due to restructuring of some community services and a requirement for further training for new staff. Of those eligible and invited to participate, 78/138 (56%) consented and were recruited to the trial (at a rate of 5.57/month). Control sites recruited n=38 compared with n=40 in intervention sites. The main reason for non-eligibility was patients not requiring six rehabilitation sessions or more (58%), and patients with cognitive and communication impairments (17%). The latter were excluded as a certain minimum level of cognitive and communication ability (ie, ability to follow a verbal or non-verbal two-stage command) was required for the intervention, which is based on cognitive interaction between practitioner and stroke survivor.

### Completion rates

**Figure 1:**
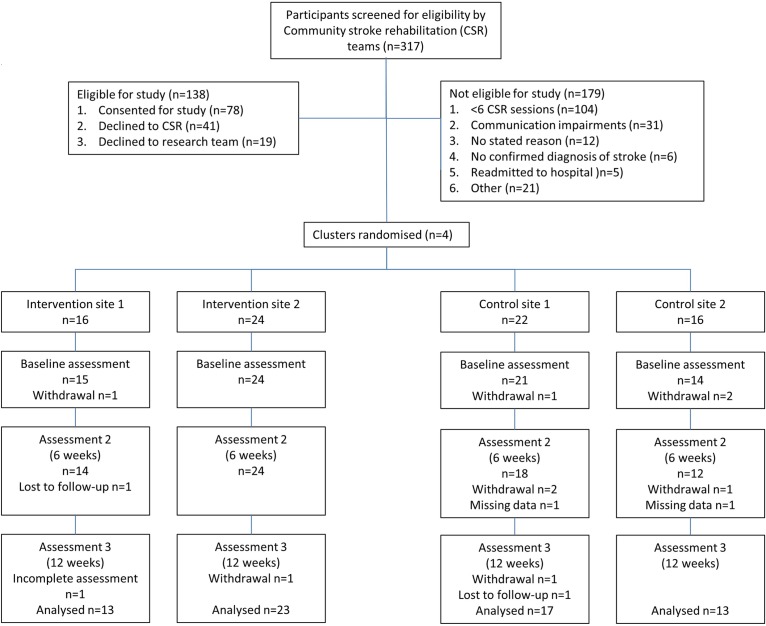
Study flow diagram.

The research protocol was successfully delivered and outcome assessors remained blinded to the intervention throughout the duration of the trial. Figure 1 shows rates of completion varied slightly between control and intervention sites. Thirty-nine participants (98%) completed baseline measures and 36 participants completed week 12 outcome measures (90%) in intervention sites, compared with 35 (92%) completing baseline outcomes and 30 (79%) completing week 12 outcomes measures in control sites. Reasons for withdrawal included ill health and change in family circumstances, with only three cases of withdrawal due to burden of outcome measurement (nature of the questions (n=1) and the volume of questions (n=2)).

### Randomisation

#### Participant characteristics

Table 2 shows an even distribution of men and women in intervention sites, but more men took part in the control sites. Days post-stroke data were missing in 8/78 participants. Of note is the wide variation in the length of time since stroke onset. Demographic variables including ethnicity and social circumstances were comparable between intervention and control sites, with the exception of age. Baseline data were complete for 74 (95%) out of 78 participants (95% CI 87% to 99%), with no significant difference between study arms (98% intervention vs 92% control, p=0.35, Fisher's exact test).

**Table 2:** Characteristics of study participants

	Intervention (n=40)	Control (n=38)
Age	61.79±16.03	68.82±10.28
Sex		
Male	20 (50%)	25 (65.8%)
Female	20 (50%)	13 (34.2%)
Time post-stroke onset (days)		
Minimum, maximum	31, 1369	17, 1105
Median (IQR)	76 (44.5–130.5)	116 (46–170.5)
Cohabitants		
Living alone	11/38 (29%)	11/37 (30%)
Spouse only	18/38 (47%)	20/37 (54%)
Others	9/38 (24%)	6/37 (16%)
Carers		
None	4/38 (10%)	6/37 (16%)
Professional	9/38 (24%)	11/37 (30%)
Family and friends only	25/38 (66%)	20/37 (54%)
Housing		
House	21/38 (55%)	23/37 (62%)
Apartment	15/38 (40%)	9/37 (24%)
Other	2/38 (5%)	5/37 (14%)
Ethnicity		
White British	17/38 (45%)	19/37 (51%)
Other White	3/38 (8%)	8/37 (22%)
Black Caribbean	10/38 (26%)	6/37 (16%)
Other	8/38 (21%)	4/37 (11%)
NEADL	29.89±14.38	30.78±17.01
HADS-A	7.54±5.27	7.43±5.10
HADS-D	6.90±4.22	7.11±3.44
SAQOL mean	3.37±0.77	3.25±0.81
SAQOL physical	3.40±0.87	3.05±1.05
SAQOL communication	4.00±1.08	4.09±0.90
SAQOL psychological	3.05±1.00	3.01±1.01
SF-12 physical	34.00±8.53	30.86±10.10
SF-12 mental	46.84±12.57	40.96±14.24
SSEQ	25.95±8.64	23.51±9.72

Values are proportion (percentage) or mean±SD, unless otherwise indicated.

HADS-A, Hospital Anxiety and Depression Scale—Anxiety scores; HADS-D, Hospital Anxiety and Depression Scale—Depression scores; NEADL, Nottingham Extended Activities of Daily Living Scale; SAQOL, Stroke and Aphasia Quality of Life scores; SF-12, Short Form 12 questionnaire; SSEQ, Stroke Self-Efficacy Questionnaire.

### Clinical outcomes

Table 3 shows means and SDs for all outcomes at each time point (baseline, 6 weeks and 12 weeks). Table 3 shows clinical data at 12 weeks completed for 66/78 participants (83%; 95% CI 75% to 92%), and there was no significant difference in outcomes between the arms of the trial for this (p=0.22, Fisher's exact test). The modelling revealed no significant difference between intervention and controls on any outcome that was tested, although the intervention sites showed more consistent improvement in self-efficacy (SSEQ) and functional capacity (NEADL) than did control sites (table 4). If the intervention is aimed at changing self-efficacy and confidence to self-manage, then functional capacity, which measures actual performance, could be a feasible clinical endpoint in a future fully powered trial.

**Table 3:** Means and SDs of outcomes at all time points

Outcome	Intervention group (n=2)			Control group (n=2)		
	Baseline	6 weeks	12 weeks	Baseline	6 weeks	12 weeks
NEADL	29.9±14.4	32.6±16.4	35.5±16.9	30.8±17.0	31.5±18.5	32.1±19.0
HADS-A*	7.5±5.3	7.5±4.9	6.6±5.3	7.4±5.1	7.3±4.9	7.4±4.5
HADS-D*	6.9±4.2	7.1±4.5	7.1±4.3	7.1±3.4	8.2±4.1	8.1±4.1
SAQOL mean	3.4±0.8	3.3±0.8	3.4±0.8	3.2±0.8	3.2±0.7	3.1±0.8
SAQOL physical	3.4±0.9	3.3±1.0	3.4±1.0	3.1±1.1	3.1±1.0	3.0±1.1
SAQOL communication	4.0±1.1	4.0±1.0	4.2±1.1	4.1±0.9	3.9±1.0	4.0±0.9
SAQOL psychological	3.1±1.0	3.0±1.0	3.1±1.1	3.0±1.0	3.0±0.9	2.8±0.9
SF-12 physical	34.0±8.5	34.9±10.0	36.3±10.8	30.9±10.1	31.6±7.0	33.1±8.8
SF-12 mental	46.8±12.6	45.5±11.8	46.1±10.7	41.0±14.2	44.7±13.1	42.8±11.9
SSEQ	25.9±8.6	25.7±9.4	26.4±9.0	23.5±9.7	21.3±9.2	21.5±10.6

*High scores on HADS indicate worse morbidity, for all other scales this is reversed.

HADS-A, Hospital Anxiety and Depression Scale—Anxiety scores; HADS-D, Hospital Anxiety and Depression Scale—Depression scores; NEADL, Nottingham Extended Activities of Daily Living Scale; SAQOL, Stroke and Aphasia Quality of Life scores; SF-12, Short Form 12 questionnaire; SSEQ, Stroke Self-Efficacy Questionnaire.

**Table 4:** Outcomes analysis

Outcome	Difference at 12 weeks	Change from baseline	Change adjusted for age	Multilevel model		
				Change at 6 weeks, adjusted for age	Change at 12 weeks, adjusted for age	Composite p value
NEADL	3.47	4.37	3.77	2.89	4.51	0.14
HADS-A*	−0.85	−0.61	−0.23	−0.06	−0.45	0.87
HADS-D*	−0.96	−0.55	−0.38	−0.93	−0.59	0.36
SAQOL mean	0.26	0.05	0.02	0.02	0.05	0.91
SAQOL physical	0.32	−0.10	−0.09	−0.03	−0.08	0.87
SAQOL communication	0.15	0.17	0.14	0.13	0.16	0.52
SAQOL psychological	0.26	0.15	0.08	0.04	0.14	0.72
SF-12 physical	3.13	−0.31	−0.37	0.61	−0.07	0.91
SF-12 mental	3.36	−1.16	−1.77	−3.92	−2.20	0.31
SSEQ	4.83	1.91	1.11	2.20	2.17	0.30

Values are expressed as mean differences between intervention and control sites. Output from the multilevel model comparing changes (adjusted for age) across collected outcome measures.

*High scores on HADS indicate worse morbidity, for all other scales this is reversed.

NEADL, Nottingham Extended Activities of Daily Living Scale; HADS-A, Hospital Anxiety and Depression Scale—Anxiety scores; HADS-D, Hospital Anxiety and Depression Scale—Depression scores; SAQOL, Stroke and Aphasia Quality of Life scores; SF-12, Short Form 12 questionnaire; SSEQ, Stroke Self-Efficacy Questionnaire.

### Sample size calculation for a definitive study

A sample size calculation for a future cluster-randomised controlled trial can be based on the NEADL at 12 weeks with MCIDs suggested as 6.1. The mean (SD) for NEADL was 35.5 (16.86) in the intervention group and 32.1 (19.05) in the control group, and Pearson correlation between baseline and 12-week follow-up NEADL was 0.78. Sites in this study were similar for NEADL apart from one site, which had a lower mean (but this seems to have been driven by just two participants); therefore, we are not able to make a precise estimate of intraclass correlation for future studies, though it appears to be small. If we assume intraclass correlation of zero in the sample size calculation, this effectively uses a calculation for parallel-arms randomised controlled trials, and the MCID for NEADL would require 137 in each arm.^34^ Assuming a pessimistic completion rate at 12 weeks of 75%, the lower end of the CI from this study's data, this requires consenting 183 participants per arm for NEADL, which implies allocating nine sites per arm for NEADL alone.

### Resources and costs of the intervention

Rehabilitation records were available for 73 patients. Total rehabilitation inputs were similar in the two control sites (24 therapy hours per patient). However, a difference was found between the two intervention sites (20.1 vs 50.7 therapy hours). Intervention sites reported a proportionately higher use of therapy assistants than control site (table 5). Costs of patient-facing time ranged from £600 in the low resource use intervention site to £1667 in the high resource use intervention site. The costs of the two control sites were similar (£754 and £763). Total costs for control sites (mean of two sites) ranged from £930 to £1459, depending on the assumptions made about the ratios of patient-facing to patient-related non-face-to-face costs. The equivalent range for the low resource use intervention site was £721–£1103, and for the high resource use intervention site it was £1987–£3012 (table 5).

**Table 5:** Resources and costs* used in delivering rehabilitation in the four sites

		Intervention sites		Control sites	
		Cluster 1	Cluster 2	Cluster 3	Cluster 4
Number of patients with therapy records		15	23	22	13
Face to face contact Mean (SD) [Minimum/Range] Mean cost (£)	Occupational Therapy	10.53 (5.28) [5/15] £347.60	5.03 (4.92) [0/17] £165.96	6.63 (8.54) [0/34.1] £218.87	6.89 (5.11) [0/14] £227.45
	Physiotherapy	14.12 (14.56) [0/55] £465.80	5.08 (5.20) [0/18] £167.51	9.02 (7.15) [0/22.1] £297.75	5.33 (5.19) [0/14] £175.92
	Speech and Language Therapy	6.33 (8.45) [0/30] £209.00	2.17 (4.91) [0/19] £71.74	4.62 (13.89) [0/65.1] £152.37	6.32 (8.43) [0/24.3] £208.66
	Therapy Assistant	25.78 (23.08) [0/76.5] £644.58	7.81 (11.10) [0/45.75] £195.20	3.77 (4.76) [0/16.2] £94.22	5.69 (7.35) [0/23] £142.31
	Total hours/cost (% by Therapy Assistant)	56.77/£1667 (45.4%/38.7%)	20.09/£600 (38.8%/32.5%)	24.04/£763 (15.6%/12.3%)	24.24/£754 (23.6%/18.9%)
Patient-related non face-to-face contact Mean cost (£) #	High estimate Medium estimate Low estimate	£1345 £672 £320	£503 £251 £121	£716 £358 £177	£683 £342 £167
Total cost: sum face-to-face and non face-to-face: high, medium and low estimates		£3012, £2339, £1987	£1103, £851, £721	£1479, £1121, £940	£1438, £1096, £921

* Unit costs, £ 2012, per hour:^33^ allied health professional (AHP), occupational therapy (OT), physiotherapy (PT), speech and language therapy (SLT)=£33; therapy assistant (TA)=£25.

# Estimates based on assumed ratios of face-to-face to non face-to-face time. High is 1:1 for OT, PT, SLT and 1:0.5 for TA; Middle is 1:0.5 for OT, PT, SLT and 1:0.25 for TA; Low is 1: 0.25 for OT, PT, SLT, and for TA.

Patient level use of other health and social services at 6-week and 12-week follow-up were available for 63 of the 73 (88%) participants; the remainder were either lost to follow-up or withdrew. There were relatively few missing data items. The only services used by more than 10% of respondents were GPs, nurses, and hospital outpatient and emergency departments (data not shown); all other services, including social care, were not accessed by more than 90% of participants. Comparisons between sites of total costs of other service utilisation revealed no significant differences between any pair of sites. However, when only stroke-related service use was considered, the other health and social service costs of patients in the low-cost intervention site were higher than in all the other sites (£1291 vs £514 in the high cost intervention site, and £529 and £898 in the two control sites).

## Discussion

This is the first study to test the feasibility of conducting a cluster-randomised controlled trial into the effectiveness of a stroke SMP integrated into post-hospital rehabilitation. Overall, the design, using a nested process evaluation, was found to be feasible and the intervention was delivered according to predetermined markers of fidelity.

Recruitment rate at 25% was higher than previous research (18%).^24^ But patients who required fewer than six sessions were the main reason for exclusion (58%). This is a limitation of this study and our previous research, but was chosen following discussion with CSR teams based on the premise that patients requiring less than six sessions would be less likely to have ongoing rehabilitation needs and would usually be managed by assessment and one-off advice. However, further research to adapt self-management interventions to be delivered in fewer number of sessions while delivering the same impact, such as that developed by Harwood *et al*, are now warranted.^35^ Participants with aphasia and other cognitive impairments were also recruited at a lower rate and previous research using provider-based stroke SMPs has included low numbers of people with aphasia.^9^ Participants were also excluded due to low mood, not engaging in therapy and social issues, and 12 potential participants were excluded with no clear reason other than they were less compliant or more challenging. We suspect there were issues of potential gate keeping and selection of ‘model’ participants for the trials illustrated in another study,^36^ which highlights the need for training to include methods and practical solutions of extending the SMP to more patients.

Outcomes measuring functional capacity (NEADL) and self-efficacy (SSEQ) showed most sensitivity to change in the intervention compared with control sites. This provides some validation of the aims of Bridges stroke SMP, which uses self-efficacy principles to facilitate a change in functional capacity and self-management. Functional capacity and mood have been shown to be closely associated with self-efficacy post-stroke, but the causal relationship has not been established.^11^ ^37^ However, we suggest a measure of functional capacity such as the NEADL as a primary outcome with secondary measures of mood, quality of life and self-efficacy, is warranted in future self-management trials. At least 18 clusters would be required recruiting 20 participants per site to evaluate effectiveness of this stroke SMP in a full trial.

A number of economic findings were relevant to a full trial. In particular, the resource implications of the intervention appeared very different in the two sites. The composition of teams, particularly the ratio of professional to support staff also require further evaluation. The tool for collecting data on other service use worked well, but the burden on participants might have been reduced by concentrating on services (including GP, nurse, A&E, nurse inpatient) used most frequently, and only on those that were stroke-related. The SF-12 scores were not significantly different between groups, so QALYs were not calculated, although a larger trial may identify differences.

The quality of training given to clinicians in the intervention sites was central to the delivery of the SMP as intended, but was more labour intensive than expected due to high staff turnover. However, compared with recent large-scale trials of provider-based SMPs,^38–40^ clinicians from the intervention sites engaged in training, and enacted behaviours aligned with predetermined markers of self-management support. Nonetheless, training costs are a major consideration for SMP implementation, and less costly methods of training, such as online resources and peer learning utilising SMP champions, could be employed in a full trial.

Overall, the study was completed with minimal data lost to follow-up, and the trial design could be replicated in a larger definitive trial. By reducing the number of sessions required, addressing accessibility of the workbook and adapting the intervention for people with cognitive impairments, recruitment rates could increase further. Given these recommendations, our results support the need for conducting further research in this area, and provide data to support the design of a definitive trial.

## References

[1] Feigin VL, Forouzanfar MH, Krishnamurthi R, Global Burden of Diseases, Injuries, and Risk Factors Study 2010 (GBD 2010) and the GBD Stroke Experts Group. Global and regional burden of stroke during 1990–2010: findings from the Global Burden of Disease Study. Lancet 2014;383:245–54. 10.1016/S0140-6736(13)61953-4 24449944 PMC4181600

[2] Bray B, Ayis S, Campbell J Associations between the organisation of stroke services, process of care, and mortality in England: prospective cohort study. BMJ 2013;346:f2827 10.1136/bmj.f2827 23667071 PMC3650920

[3] Langhorne P, Bernhardt J, Kwakkel G Stroke rehabilitation. Lancet 2011;377:1693–702. 10.1016/S0140-6736(11)60325-5 21571152

[4] The Intercollegiate Stroke Working Party, Royal College of Physicians. National Sentinel Stroke Audit. Organisational Audit 2010 Public Report for England, Wales and Northern Ireland, 2010 https://www.rcplondon.ac.uk/sites/default/files/national-sentinel-stroke-audit-2010-public-report-and-appendices_0.pdf (accessed 15 Sep 2014).

[5] McKevitt C, Fudge N, Redfern J Self-reported long-term needs after stroke. Stroke 2011;42:1398–403. 10.1161/STROKEAHA.110.598839 21441153

[6] Ayerbe L, Ayis S, Rudd AG Natural history, predictors, and associations of depression 5 years after stroke. The South London Stroke Register. Stroke 2011;42:1907–11. 10.1161/STROKEAHA.110.605808 21566241

[7] Boden-Albala B, Litwak E, Elkind MSV Social isolation and outcomes post stroke. Neurology 2005;64:1888–92. 10.1212/01.WNL.0000163510.79351.AF 15955939

[8] Saka O, Mcguire A, Wolfe C Cost of stroke in the United Kingdom. Age Ageing 2009;38:27–32. 10.1093/ageing/afn281 19141506

[9] Kendall E, Catalano T, Kuipers P Recovery following stroke: the role of self-management education. Soc Sci Med 2007;64:735–46. 10.1016/j.socscimed.2006.09.012 17079060

[10] O'Neill D, Horgan F, Hickey A Stroke is a chronic disease with acute events. BMJ 2008;336:461 10.1136/bmj.39500.434086.1F PMC2258352 18309967

[11] Jones F, Riazi A Systematic review of self-efficacy and stroke. Disabil Rehabil 2010;33:797–810. 10.3109/09638288.2010.511415 20795919

[12] de Silva D Helping people help themselves: a review of the evidence considering whether it is worthwhile to support self-management. The Health Foundation, 2011 http://www.health.org.uk/media_manager/public/75/publications_pdfs/Helping%20people%20help%20themselves.pdf (accessed 15 Sep 2014).

[13] Newbronner L, Chamberlain R, Borthwick M Sustaining and spreading self-management support: lessons from co-creating health phase 2. The Health Foundation, 2013 http://www.health.org.uk/public/cms/75/76/313/4422/Sustaining%20and%20spreading%20self-management%20support.pdf?realName=mjapW3.pdf (accessed 15 Sep 2014).

[14] Newman S, Steed L, Mulligan K Self-management interventions for chronic illness. Lancet 2004;364:1523–37. 10.1016/S0140-6736(04)17277-2 15500899

[15] Kennedy A, Rogers A, Bower P Support for self care for patients with chronic disease. BMJ 2007;335:968–70. 10.1136/bmj.39372.540903.94 17991978 PMC2071971

[16] National Voices. Supporting self-management: summarising evidence from systematic reviews 2014 http://www.nationalvoices.org.uk/sites/www.nationalvoices.org.uk/files/supporting_self-management.pdf (accessed 15 Sep 2014).

[17] Department of Health. A new ambition for stroke: a consultation on a national strategy 2007 http://webarchive.nationalarchives.gov.uk/20130107105354/http://www.dh.gov.uk/prod_consum_dh/groups/dh_digitalassets/documents/digitalasset/dh_081059.pdf (accessed 15 Sep 2014).

[18] Intercollegiate Working Party for Stroke. National Clinical Guidelines for Stroke. 4th edn Royal College of Physicians, 2012 https://www.rcplondon.ac.uk/sites/default/files/national-clinical-guidelines-for-stroke-fourth-edition.pdf</authors> (accessed 15 Sep 2014).

[19] Lennon S, McKenna S, Jones F Self-management programmes for people post stroke: a systematic review. Clin Rehabil 2013;27:867–78. 10.1177/0269215513481045 23543340

[20] Cadilhac DA, Hoffman S, Kilkenny M A phase II multicentered, single-blind, randomized, controlled trial of the stroke self-management program. Stroke 2011;42:1673–9. 10.1161/STROKEAHA.110.601997 21493910

[21] Davidson B, Howe T, Worrall L Social participation for older people with aphasia: the impact of communication disability on friendships. Top Stroke Rehabil 2008;15:325–40. 10.1310/tsr1504-325 18782736

[22] Jones F, Mandy A, Partridge C Changing self-efficacy in individuals following first stroke: preliminary study of a novel self-management intervention. Clin Rehabil 2009;23:522–33. 10.1177/0269215508101749 19403556

[23] Craig P, Dieppe P, Macintyre S Medical Research Council Guidance. Developing and evaluating complex interventions: the new Medical Research Council guidance. BMJ 2008;337:a1655 http://www.mrc.ac.uk/documents/pdf/complex-interventions-guidance/ (accessed 15 Sep 2014). 10.1136/bmj.a1655 18824488 PMC2769032

[24] McKenna S, Jones F, Glenfield P Bridges self-management programme for people with stroke in the community: a feasibility randomised controlled trial. Int J Stroke 2015;10:697–704. http://onlinelibrary.wiley.com/journal/10.1111/(ISSN)1747-4949/earlyview (accessed 15 Sep 2014). 10.1111/ijs.12195 24256085

[25] Newman S, Steed L, Mulligan K Chronic physical illness: self-management and behavioural interventions. Berkshire: Open University Press, 2009.

[26] Bandura A The nature and structure of self-efficacy. In: Bandura A, ed.. Self-efficacy: the exercise of control. New York, NY: WH Freeman and Company, 1997:37–78.

[27] Hilari K, Byng S, Lamping DL Stroke and Aphasia Quality of Life Scale-39 (SAQOL-39): evaluation of acceptability, reliability, and validity. Stroke 2003;34:1944–50. 10.1161/01.STR.0000081987.46660.ED 12855827

[28] Nouri FM, Lincoln NB An extended activities of daily living scale for stroke patients. Clin Rehabil 1987;1:301–5. 10.1177/026921558700100409

[29] Jones F, Partridge C, Reid F The Stroke Self-Efficacy Questionnaire: measuring individual confidence in functional performance after stroke. J Clin Nurs 2008;17:244–52. 10.1111/j.1365-2702.2008.02333.x 18578800

[30] Riazi A, Aspden T, Jones F Stroke Self-efficacy Questionnaire: a Rasch-refined measure of confidence post stroke. J Rehabil Med 2014;46:406–12. 10.2340/16501977-1789 24658341

[31] Aben I, Verhey F, Lousberg R Validity of the beck depression inventory, hospital anxiety and depression scale, SCL-90, and hamilton depression rating scale as screening instruments for depression in stroke patients. Psychosomatics 2002;43: 386–93. 10.1176/appi.psy.43.5.386 12297607

[32] Gray AM, Rivero-Arias O, Clarke PM Estimating the association between SF-12 responses and EQ-5D utility values by response mapping. Med Decis Making 2006;26:18–29. 10.1177/0272989X05284108 16495197

[33] Curtis L Unit costs of health and social care. The Personal Social Research Unit, 2012 http://www.pssru.ac.uk/project-pages/unit-costs/2012/index.php

[34] Wang D, Bakhai A Clinical Trials. London: Remedica, 2006.

[35] Harwood M, Weatherall M, Talemaitoga A Taking charge after stroke: promoting self-directed rehabilitation to improve quality of life—a randomized controlled trial. Clin Rehabil 2012;26:493–501. 10.1177/0269215511426017 22087047

[36] Lake A, Staiger P Seeking the views of health professionals on translating chronic disease self-management models into practice. Patient Educ Couns 2010;79:62–8. 10.1016/j.pec.2009.07.036 19733460

[37] Korpershoek C, van der Bijl J, Hafsteinsdóttir TB Self-efficacy and its influence on recovery of patients with stroke: a systematic review. J Adv Nurs 2011;67:1876–94. 10.1111/j.1365-2648.2011.05659.x 21645040

[38] Coulter A, Ellins J Improving self-care. In: Coulter A, Ellins J Patient-focused interventions. A review of the evidence. London: The Health Foundation, 2006:85–141.

[39] Greenhalgh T, Campbell-Richards D, Vijayaraghaven S New models of self-management education for minority ethnic groups: pilot randomised trial of a story-sharing intervention. J Health Serv Res Policy 2011;16:28–36. 10.1258/jhsrp.2010.009159 20739577

[40] Kennedy A, Rogers A, Chew-Graham C Implementation of a self-management support approach (WISE) across a health system: a process evaluation explaining what did and did not work for organisations, clinicians and patients. Implement Sci 2014;9:129 10.1186/s13012-014-0129-5 25331942 PMC4210530

